# Rheumatoid arthritis-associated rheumatoid factors post-COVID-19

**DOI:** 10.3389/fimmu.2025.1553540

**Published:** 2025-02-13

**Authors:** Adam H. Titi, Braedon T. Krisko, S. Janna Bashar, Ryan R. Adyniec, Maxwell H. Parker, Nat F. Murren, Courtney B. Myhr, Miriam A. Shelef

**Affiliations:** ^1^ Department of Medicine, University of Wisconsin-Madison, Madison, WI, United States; ^2^ William S. Middleton Memorial Veterans Hospital, Madison, WI, United States

**Keywords:** COVID-19, rheumatoid arthritis, rheumatoid factor, autoimmunity, autoantibody

## Abstract

**Objective:**

Rheumatoid factors (RFs) are a hallmark of rheumatoid arthritis but also arise in infections, including COVID-19. Moreover, infections, again including COVID-19, are associated with rheumatoid arthritis development, positioning RFs as a potential link between infection and rheumatoid arthritis. RFs traditionally have been thought to be relatively uniform in their reactivity across conditions apart from some increased reactivity in rheumatoid arthritis. Recently, however, IgG RFs that bind citrulline- and homocitrulline-containing IgG epitopes were identified in rheumatoid arthritis, but not other autoimmune diseases, whereas IgM RFs that bind specific native linear IgG epitopes were found uniquely post-COVID-19. The objective of this study was to determine if rheumatoid arthritis-associated RFs develop post-COVID-19 in order to provide new insights into post-infection immune tolerance loss.

**Methods:**

COVID-19 convalescent, rheumatoid arthritis, and control sera (n=20) were used in enzyme-linked immunosorbent assay to evaluate IgG, IgM, and IgA binding to eight IgG1-derived peptides in their native, citrulline-containing, and homocitrulline-containing forms. Antibody levels were compared by Kruskal-Wallis test with Dunn’s multiple comparisons test, and the number of participants with binding greater than all controls was compared by Fisher’s exact test.

**Results:**

IgG binding to seven of the eight IgG1-derived peptides was increased in a citrulline- or homocitrulline-specific manner only in rheumatoid arthritis. IgA binding was increased to five of eight IgG1-derived peptides in a citrulline- or homocitrulline-specific manner in rheumatoid arthritis and to one homocitrulline-containing peptide post-COVID-19. More post-COVID-19 participants than controls had elevated IgG or IgA binding to two IgG1-derived peptides in a homocitrulline-specific manner.

**Conclusion:**

Rheumatoid arthritis-associated RFs are primarily restricted to rheumatoid arthritis, but some individuals post-COVID-19 generate moderate levels of a few rheumatoid arthritis-associated RFs, especially of the IgA isotype and homocitrulline-reactive. These findings refine our understanding of RFs, provide novel insights into loss of immune tolerance post-infection, and reveal new possibilities for biomarker development in preclinical rheumatoid arthritis.

## Introduction

In rheumatoid arthritis, two hallmark antibodies commonly arise: anti-citrullinated protein antibodies (ACPAs) and rheumatoid factors (RFs). ACPAs, which are clinically detected with the diagnostic anti-cyclic citrullinated peptide (CCP) test, bind citrulline-containing epitopes in a manner that can be specific for individual targets or multi-reactive with many citrullinated and homocitrullinated epitopes (1, 2). RFs are antibodies with varying degrees of multi-reactivity (3) that bind the Fc region of IgG and are also used diagnostically for rheumatoid arthritis (4). Both of these antibody types appear years prior to the onset of rheumatoid arthritis, typically first ACPAs and then RFs (5, 6). A positive anti-CCP test is highly specific for rheumatoid arthritis (7), but RFs can develop in a variety of conditions, including COVID-19 (8, 9) and other infections (10–12). Moreover, several infections, including COVID-19, are associated with rheumatoid arthritis development (13–15). Thus, infection may trigger a loss of immune tolerance for self-antigens in some individuals, but the steps of immune tolerance loss post-infection are largely unknown.

RFs are canonically known to bind two conformational epitopes of IgG Fc with some additional reactivities in rheumatoid arthritis (16–19). Recently, however, IgG RFs with reactivity to citrulline- and homocitrulline-containing linear IgG peptides were identified in rheumatoid arthritis but not in other autoimmune diseases (20, 21). In contrast, RFs, primarily of the IgM isotype, that react with three native linear IgG epitopes were identified in COVID-19 but not in rheumatoid arthritis or other conditions (9). These findings suggest that unique RFs exist in different conditions. However, rheumatoid arthritis-associated RFs have not yet been evaluated in the context of infection.

The purpose of this study was to determine if rheumatoid arthritis-associated RFs develop post-COVID-19 in order to refine our understanding of RFs, provide insights into immune tolerance loss post-infection, and reveal novel biomarker possibilities.

## Materials and methods

### Human subjects

This study was conducted in accordance with the Declaration of Helsinki and was approved by the Institutional Review Board at the University of Wisconsin (UW). Informed consent was obtained for experimentation with human subjects. Serum from participants ~5 weeks post-COVID-19 symptom resolution (positive SARS-CoV-2 PCR test in the spring of 2020) with no known rheumatoid arthritis per medical record review were obtained from the UW COVID-19 Convalescent Biorepository (22). Serum from age- and gender-matched participants with rheumatologist-diagnosed rheumatoid arthritis or no autoimmune or inflammatory disease (controls) were obtained from the UW Rheumatology Biorepository (23). Control and rheumatoid arthritis sera were collected prior to 2019. Rheumatoid arthritis subjects had clinical test results for anti-CCP and RF >2x the upper limit of normal.

### Enzyme-linked immunosorbent assay

ELISA was performed using native, citrulline (B)-containing, homocitrulline (J)-containing, and dually modified (BJ) peptides beginning at amino acids 11, 80, 131, 167, 202, 219, 236, and 289 of the constant region of the heavy chain of IgG1 (Uniprot P01857) as described in full previously (21) with a few modifications. In brief, 96-well plates were coated with 5 µg/ml streptavidin (Thermo Scientific Pierce, Waltham, MA) for one hour at room temperature, washed with PBS, and incubated with 1 µM biotinylated peptides in PBS or PBS alone (uncoated wells) for one hour at room temperature. After washing, plates were blocked with blocking solution (5% non-fat dehydrated milk in 0.2% Tween 20 in PBS) for 3 hours at room temperature, and then incubated with serum (diluted 1:100 in blocking solution for IgA and IgM and 1:200 for IgG) overnight at 4°C. Plates were then washed, incubated for one hour at room temperature with mouse monoclonal anti-human IgG (clone JDC-10), goat anti-human IgM, or goat anti-human IgA conjugated to horseradish peroxidase (Southern Biotechnology, Birmingham, AL, USA) diluted 1:5000 in blocking solution. Plates were washed, developed with 3,3′,5,5′-tetramethylbenzidine substrate solution (Thermo Scientific Pierce), and the reaction was stopped using 0.18M sulfuric acid after 15 minutes for IgG and IgA and 5 minutes for IgM. Absorbance (450-562nm) was read on a FilterMax F3 spectrophotometer (Molecular Devices, San Jose, CA, USA) and absorbance values from uncoated wells were subtracted from coated wells for each sample. Washes with 0.2% Tween 20 in PBS (six after peptide, serum, and secondary antibody incubations) were performed using a BioTek 405 TS microplate washer (Agilent, Santa Clara, CA, USA). Peptide sequences were previously published (9, 21) apart from the following: IgG1-131J, JDTLMISRTPEVTCVV; IgG1-131BJ, JDTLMISBTPEVTCVV; IgG1-236, PSRDELTKNQVSLTCLVK; IgG1-236B, PSBDELTKNQVSLTCLVK.

Anti-CCP IgG was detected using a commercial ELISA (EDIA ™ anti-CCP kit, FCCP100, Svar Life Science, Malmö, Sweden) according to the manufacturer’s instructions.

### Statistical analysis

A Kruskal Wallis test with Dunn’s multiple comparisons test was used to compare antibody levels, a Mann-Whitney test to compare the number of bound peptides, and a Fisher’s exact test to compare the number of participants with antibody levels higher than all controls with p < 0.05 considered significant (Prism, Graphpad, San Diego, CA).

## Results

**Figure 1:**
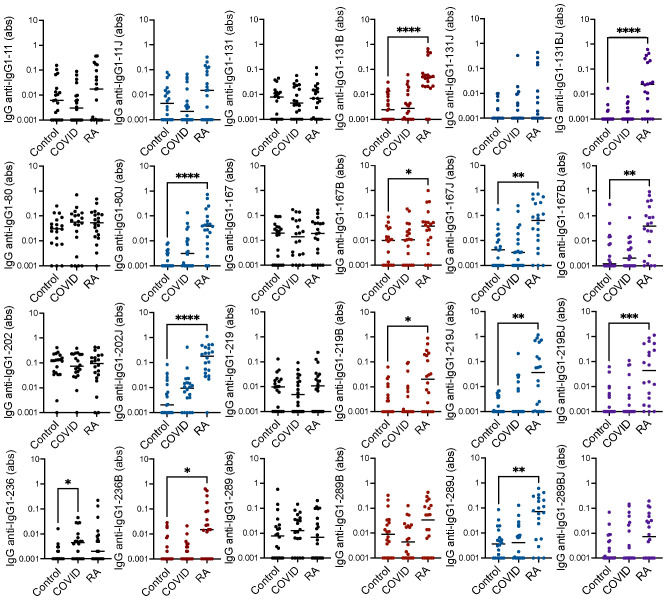
High IgG binding to most citrulline- and homocitrulline-containing IgG1 peptides in rheumatoid arthritis but not post-COVID-19. Serum IgG from matched seropositive rheumatoid arthritis (RA), COVID-19 convalescent, and control participants (n=20) was quantified by ELISA for binding to native, citrulline (B)-containing, homocitrulline (J)-containing, and dually modified forms of IgG1 peptides beginning at amino acids 11, 80, 131, 167, 202, 219, 236 and 289. To determine if antibody levels were increased in RA or post-COVID-19, absorbance (abs) values were compared for RA and COVID-19 convalescent participants versus controls by Kruskal Wallis test with Dunn’s multiple comparisons test. Lines indicate medians and *p<0.05, **p<0.01, ***p<0.001, ****p<0.0001. Comparisons with a p value ≥ 0.05 were not marked.

To determine if individuals post-COVID-19 generate rheumatoid arthritis-associated RFs, we evaluated IgG binding by ELISA to eight peptides derived from the constant region of the heavy chain of IgG1 in their native form, in a form in which arginines are replaced by citrullines, in a form in which lysines are replaced by homocitrullines, and in a dually modified form for age- and gender-matched COVID-19 convalescent, rheumatoid arthritis, and control sera. We included peptides that we previously demonstrated were bound by IgG in rheumatoid arthritis (9, 21) as well as new peptides: homocitrulline-containing versions of IgG1-131 (which is bound by IgM in its native form post-COVID-19 and by IgG in its citrulline-containing form in rheumatoid arthritis (9)) and IgG1-236 (which includes a previously described peptide starting at position 238 of IgG1 that is highly bound by IgM post-COVID-19 (9)). As shown in **Figure 1** and largely consistent with previous results (9, 21), IgG binding to 12 of the 16 citrulline- or homocitrulline-containing IgG1 peptides, but none of the native peptides (except for a trend towards increased binding to native IgG1-236, p = 0.07), was increased in the rheumatoid arthritis subjects compared to controls. The only peptide with increased IgG binding post-COVID-19 compared to controls was the native form of IgG1-236.

**Figure 2:**
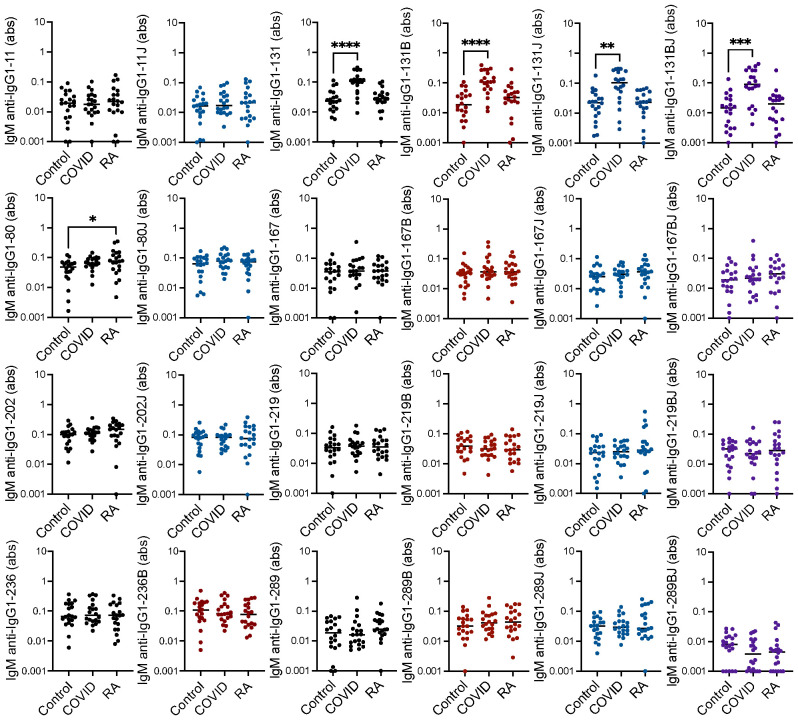
Increased IgM binding to one IgG1 peptide in rheumatoid arthritis and IgG1-131 peptides post-COVID-19. Serum IgM from matched seropositive rheumatoid arthritis (RA), COVID-19 convalescent, and control participants (n=20) was quantified by ELISA for binding to native, citrulline (B)-containing, homocitrulline (J)-containing, and dually modified forms of IgG1 peptides beginning at amino acids 11, 80, 131, 167, 202, 219, 236 and 289. To determine if antibody levels were increased in RA or post-COVID-19, absorbance (abs) values were compared for RA and COVID-19 convalescent participants versus controls by Kruskal Wallis test with Dunn’s multiple comparisons test. Lines indicate medians and *p<0.05, **p<0.01, ***p<0.001, ****p<0.0001. Comparisons with a p value ≥ 0.05 were not marked.

We previously detected only limited IgM binding to linear IgG1 peptides in rheumatoid arthritis (20). However, since COVID-19-associated RFs are primarily IgM RFs (9), we hypothesized that some IgM binding might occur post-COVID-19 to the peptides bound by rheumatoid arthritis-associated RFs. Thus, we performed ELISAs to detect IgM binding to the peptides noted above. Only IgG1-131, in a citrulline- and homocitrulline-independent manner, was highly bound by IgM post-COVID-19, likely due to the largely unaltered binding motif for the COVID-19-associated RF (9). Also, one native IgG1-derived peptide had slightly increased IgM binding in rheumatoid arthritis (**Figure 2**).

**Figure 3:**
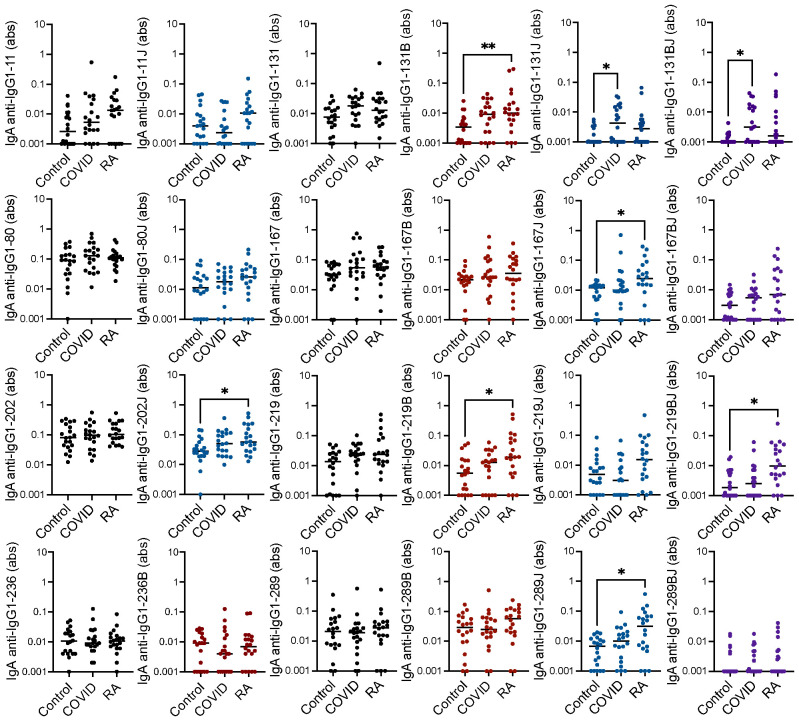
High IgA binding to some citrulline- and homocitrulline-containing IgG1 peptides primarily in rheumatoid arthritis. Serum IgA from matched seropositive rheumatoid arthritis (RA), COVID-19 convalescent, and control participants (n=20) was quantified by ELISA for binding to native, citrulline (B)-containing, homocitrulline (J)-containing, and dually modified forms of IgG1 peptides beginning at amino acids 11, 80, 131, 167, 202, 219, 236 and 289. To determine if antibody levels were increased in RA or post-COVID-19, absorbance (abs) values were compared for RA and COVID-19 convalescent participants versus controls by Kruskal Wallis test with Dunn’s multiple comparisons test. Lines indicate medians and *p<0.05, **p<0.01. Comparisons with a p value ≥ 0.05 were not marked.

We also considered IgA binding to the IgG1 peptides, an isotype that we had not extensively evaluated previously in rheumatoid arthritis or post-COVID-19. By repeating the above ELISAs to detect IgA, we found that IgA binding to 6 of the 16 citrulline- or homocitrulline-containing IgG1 peptides was increased in rheumatoid arthritis compared to controls with no increased IgA binding to native peptides in rheumatoid arthritis (**Figure 3**). When comparing COVID-19 convalescent versus control, homocitrulline-containing IgG1-131 was more highly bound by IgA in the post-COVID-19 group with a trend towards increased binding to the native and citrulline-containing forms of IgG1-131 (p = 0.06 and p = 0.05, respectively) as well.

Together, these data suggest that IgG binding to citrulline- and homocitrulline-containing IgG peptides is increased in rheumatoid arthritis, but not post-COVID-19, with similar, although less prominent, findings for IgA. However, some COVID-19 convalescent participants appeared to have higher levels of a few of these antibodies compared to controls. Thus, we quantified the number of rheumatoid arthritis and COVID-19 convalescent participants with antibody levels greater than all controls for each peptide. As shown in **Table 1**, more rheumatoid arthritis participants than controls had high IgG binding to all eight peptides, primarily in their citrulline- or homocitrulline-containing forms. For the COVID-19 convalescent participants, more had high IgG binding than controls to two homocitrulline-containing peptides. Of note, these two peptides were highly bound in twice as many rheumatoid arthritis subjects as COVID-19 convalescent subjects. For IgA, more rheumatoid arthritis participants had high binding to five peptides, and more COVID-19 convalescent participants had high binding to three peptides than controls. However, whereas elevated IgG binding was overwhelming citrulline- and homocitrulline-specific, IgA bound to about a third of the highly bound peptides in their native form. Overall, few rheumatoid arthritis participants had increased IgM binding. For COVID-19, as expected (9), there were more participants with higher IgM binding to the IgG1-131 peptides than controls. No peptide had significantly more control participants with higher IgA, IgM, or IgG binding than COVID-19.

**Table 1:** Presence of rheumatoid arthritis-associated rheumatoid factors in rheumatoid arthritis and COVID-19 convalescent participants.

	Rheumatoid Arthritis Number (%) ^#^			COVID-19 Number (%) ^##^		
Peptide	IgM	IgG	IgA	IgM	IgG	IgA
IgG1-11	3 (15)	5 (25)*	3 (15)	1 (5)	0 (0)	3 (15)
IgG1-11J	3 (15)	6 (30)*	2 (10)	3 (15)	0 (0)	0 (0)
IgG1-80	5 (25)*	2 (10)	1 (5)	1 (5)	1 (5)	2 (10)
IgG1-80J	0 (0)	15 (75)****	2 (10)	3 (15)	7 (35)*	0 (0)
IgG1-131	0 (0)	2 (10)	3 (15)	10 (50)***	1 (5)	2 (10)
IgG1-131B	1 (5)	13 (65)****	4 (20)	10 (50)***	2 (10)	4 (20)
IgG1-131J	0 (0)	5 (25)*	3 (15)	6 (30)*	3 (15)	9 (45)**
IgG1-131BJ	1 (5)	11 (55)***	7 (35)*	8 (40)**	0 (0)	9 (45)**
IgG1-167	0 (0)	2 (10)	6 (30)*	1 (5)	3 (15)	8 (40)*
IgG1-167B	1 (5)	4 (20)	5 (25)*	2 (10)	2 (10)	5 (25)*
IgG1-167J	1 (5)	6 (30)*	11 (55)***	0 (0)	1 (5)	5 (25)*
IgG1-167BJ	1 (5)	4 (20)	8 (40)**	2 (10)	0 (0)	2 (10)
IgG1-202	1 (5)	2 (10)	1 (5)	1 (5)	0 (0)	1 (5)
IgG1-202J	2 (10)	13 (65)****	6 (30)*	0 (0)	1 (5)	2 (10)
IgG1-219	0 (0)	1 (5)	6 (30)*	1 (5)	0 (0)	4 (20)
IgG1-219B	1 (5)	7 (35)*	7 (35)*	0 (0)	2 (10)	1 (5)
IgG1-219J	4 (20)	12 (60)***	3 (15)	0 (0)	6 (30)*	0 (0)
IgG1-219BJ	5 (25)*	10 (50)***	8 (40)**	1 (5)	3 (15)	2 (10)
IgG1-236	0 (0)	3 (15)	1 (5)	1 (5)	4 (20)	2 (10)
IgG1-236B	0 (0)	7 (35)*	2 (10)	0 (0)	0 (0)	3 (15)
IgG1-289	4 (20)	0 (0)	1 (5)	3 (15)	0 (0)	1 (5)
IgG1-289B	3 (15)	1 (5)	1 (5)	2 (10)	0 (0)	1 (5)
IgG1-289J	5 (25)*	9 (45)**	11 (55)***	2 (10)	1 (5)	5 (25)*
IgG1-289BJ	3 (15)	3 (15)	3 (15)	0 (0)	2 (10)	1 (5)

^#^Number (and percentage) of the 20 participants with rheumatoid arthritis who have levels of antibody that bind to each listed IgG1-derived peptide greater than the levels in all 20 control participants.

^##^Number (and percentage) of the 20 participants five weeks post-COVID-19 who have levels of antibody that bind to each listed IgG1-derived peptide greater than the levels in all 20 control participants.

*p<0.05, **p<0.01, ***p<0.001, ****p<0.0001 for disease group compared to controls by Fisher’s exact test.

**Figure 4:**
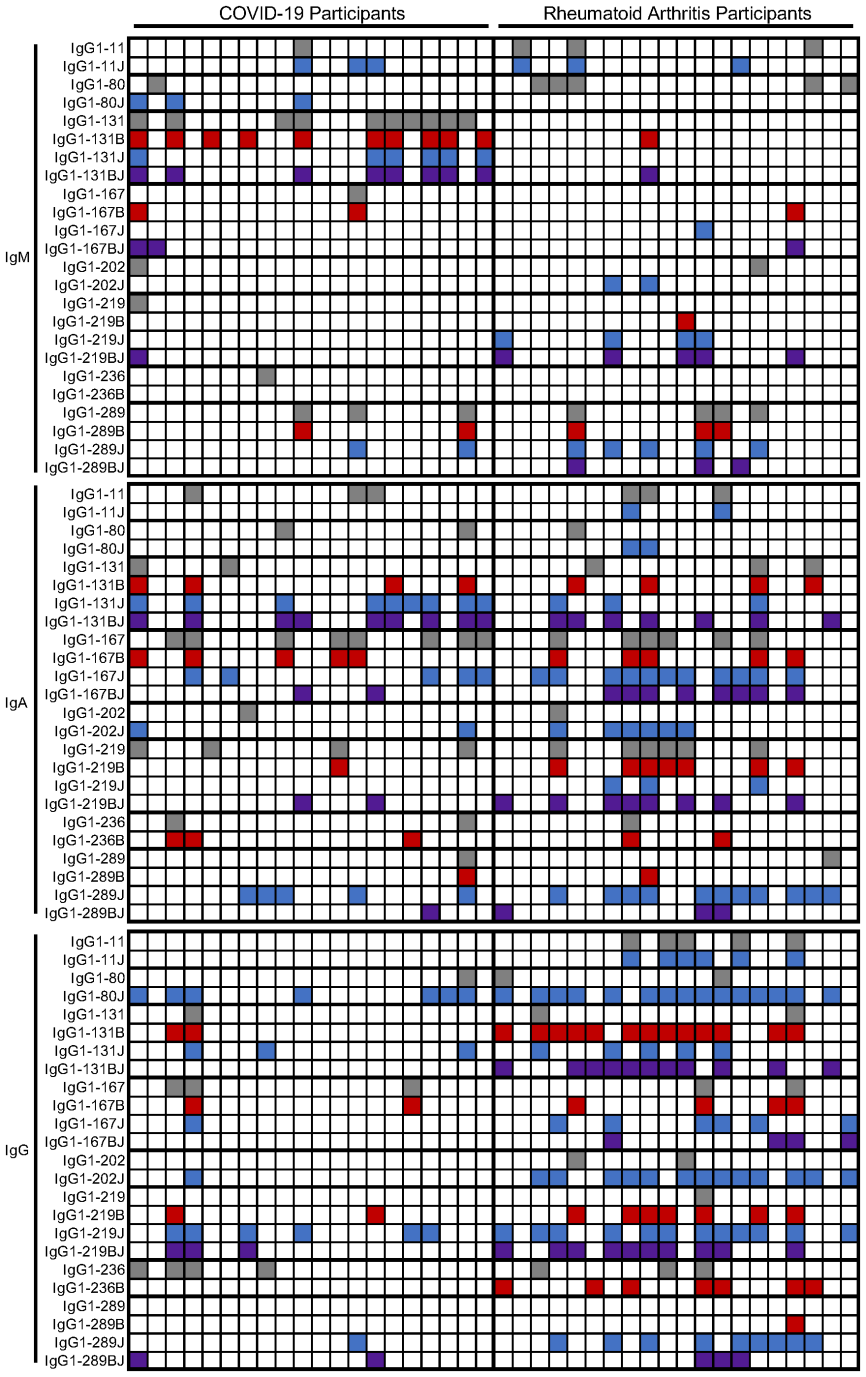
Heatmap of positivity for IgM, IgA, and IgG binding to IgG1-derived peptides in rheumatoid arthritis and post-COVID-19. Participants (columns) were marked as positive for IgM, IgA, or IgG binding to native (gray), citrulline-containing (B, red), homocitrulline-containing (J, blue), or dually modified (BJ, purple) IgG1-derived peptides (rows) based on higher binding than controls.

Individual participants varied in the number and pattern of antibody reactivities (**Figure 4**). However, there were more rheumatoid arthritis than post-COVID-19 participants with elevated IgG binding to at least one peptide (19/20 versus 12/20 subjects, p = 0.02), and rheumatoid arthritis subjects had elevated IgG against four times more peptides than post-COVID-19 participants (median 4/8 versus 1/8 peptides, p < 0.0001) in a citrulline- or homocitrulline-specific manner. There was less reactivity in general for IgA and no difference between rheumatoid arthritis and COVID-19 for both the number of participants with elevated IgA against at least one peptide (16/20 versus 14/20, respectively) or the number of peptides highly bound by IgA (median 1.5/8 versus 1/8 peptides, respectively) when evaluated in a citrulline- or homocitrulline-specific manner. Thus, while increased IgG and IgA binding to citrulline- and homocitrulline-containing IgG peptides is generally specific for rheumatoid arthritis (**Figures 1**, **3**), there is some reactivity, especially for IgA and homocitrulline-containing IgG epitopes, in some post-COVID-19 participants (**Table 1**, **Figure 4**).

**Figure 5:**
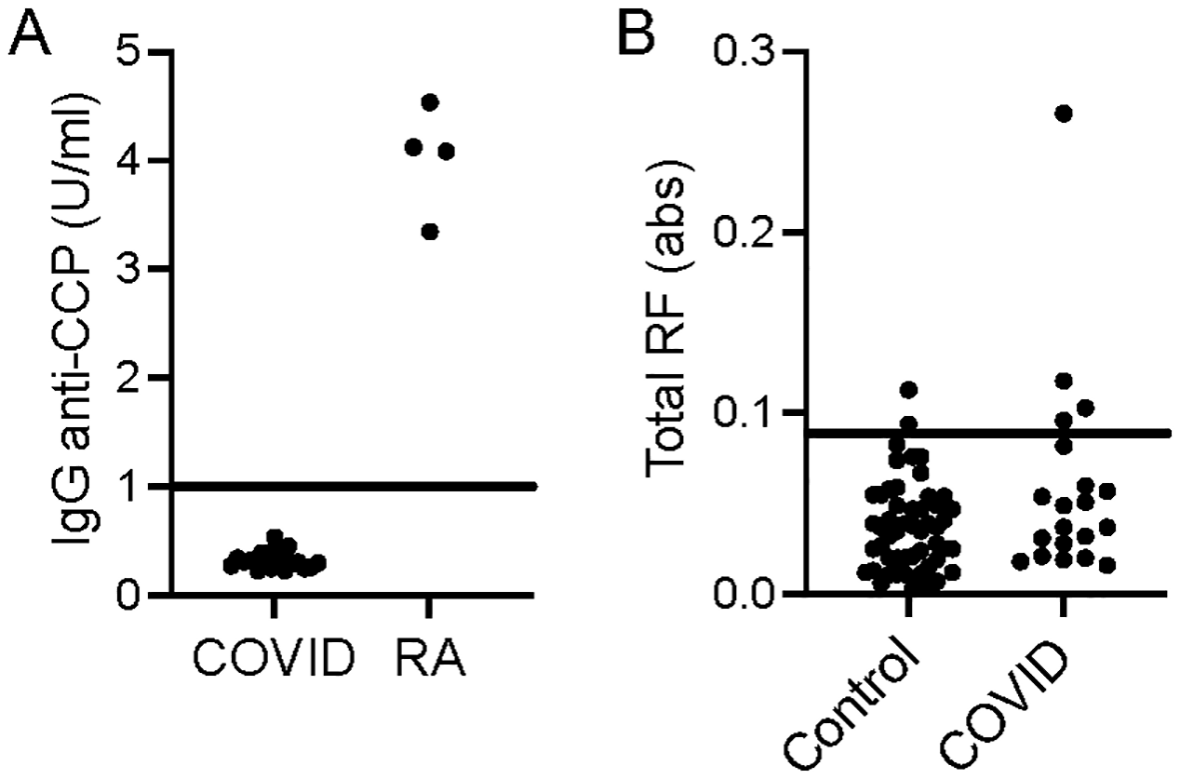
Anti-CCP IgG and traditional RF levels for the COVID-19 convalescent subjects in this study. **(A)** COVID-19 convalescent (n=20) and randomly-selected seropositive rheumatoid arthritis (n=4) sera were used in a commercial assay to detect anti-CCP IgG. **(B)** COVID-19 convalescent (n=20) and control (n=53) sera were previously assessed for traditional RF levels by IgG Fc ELISA reported in absorbance (abs). Individual values and cut-offs are depicted.

Given the binding of IgG to homocitrulline-containing IgG peptides in some COVID-19 convalescent sera and the known multi-reactivity of many ACPAs (1, 2), we evaluated if the sera contained anti-CCP IgG. Using a commercially available anti-CCP test, we found that none of the COVID-19 participants tested positive for anti-CCP IgG at the 5 week post-COVID-19 time point (**Figure 5A**). Also, we previously evaluated the COVID-19 sera used in this study for RF levels by a traditional IgG Fc ELISA as part of a larger set of samples (9). Of the 20 COVID-19 samples in this study, 20% would be considered positive for RF at the 5 week time point (**Figure 5B**). Thus, some COVID-19 convalescent participants generate classically detected RFs and antibodies against citrulline- and homocitrulline-containing IgG peptides without testing positive for the specific rheumatoid arthritis marker, anti-CCP.

## Discussion

In this manuscript we have demonstrated increased IgG and IgA binding to citrulline- and homocitrulline-containing IgG1-derived peptides, primarily restricted to rheumatoid arthritis. However, some individuals post-COVID-19 generate moderate levels of a small number of these rheumatoid arthritis-associated RFs, especially of the IgA isotype and homocitrulline-reactive, in the absence of anti-CCP positivity.

The increased binding of most citrulline- and homocitrulline-containing IgG1 peptides, but not native peptides, by IgG in most rheumatoid arthritis sera with some variability was expected (20, 21). The limited IgM binding to linear IgG1 peptides in rheumatoid arthritis is also in agreement with previous findings (9, 20) and suggests that IgM RFs in rheumatoid arthritis primarily bind conformational epitopes. The increased binding of IgA in rheumatoid arthritis to some citrulline- and homocitrulline-containing IgG1 peptides is a new finding and reveals that not only IgG, but also IgA, binds to linear citrulline- and homocitrulline-containing IgG peptides in rheumatoid arthritis. This finding is consistent with IgM RFs rising in a T cell independent manner, and class-switched citrulline- and homocitrulline-reactive IgG RFs and IgA RFs dependent upon T cell help and the preferential presentation of citrulline-containing, and potentially homocitrulline-containing, epitopes by HLA molecules with the shared epitope (24, 25).

The findings post-COVID-19 are intriguing. In the case of IgG, the levels of rheumatoid arthritis-associated IgG RFs were not increased post-COVID-19 compared to controls (**Figure 1**), but more post-COVID-19 participants than controls had elevated IgG binding, albeit at a moderate level, of two homocitrulline-containing IgG1 peptides (**Table 1**). One possible interpretation of this small study is that some of these RFs are simply not specific to rheumatoid arthritis. An alternative possibility is that high levels of these IgG RFs are specific for rheumatoid arthritis, but some rise moderately upon SARS-CoV-2 infection as part of post-viral loss of immune tolerance. Interestingly, homocitrulline-containing IgG1-80 and IgG1-219, the two peptides bound by IgG in more post-COVID-19 participants than controls, were previously shown to be bound by several monoclonal multi-reactive ACPAs (21); yet, our subjects do not have anti-CCP IgG. Thus, homocitrulline-containing IgG1-80 and IgG1-219 could be early antibody targets prior to the development of multi-reactive, CCP-binding ACPAs in a rheumatoid arthritis-related pathway of autoimmunity. If true, then these antibodies could potentially serve as novel biomarkers in preclinical rheumatoid arthritis.

The IgA RFs appear to be less specific for rheumatoid arthritis than IgG RFs. While there was increased IgA binding to citrulline- and homocitrulline-containing IgG1 peptides in rheumatoid arthritis, fewer peptides had increased binding than in the case of IgG. Also, unlike for IgG, there was increased binding by IgA to one homocitrulline-containing peptide post-COVID-19, IgG1-131, although the trend towards increased binding to other forms of the peptide suggest only partial homocitrulline-specificity. Further, there was little difference between COVID-19 and rheumatoid arthritis when evaluating the number of participants with IgA RFs. These data suggest either a similar mechanism for the rise of these IgA RFs post-COVID-19 and in rheumatoid arthritis or, perhaps, a virus-induced mechanism for their development early in a progression towards rheumatoid arthritis, at which point they are present at higher levels.

In general, the mechanisms by which SARS-CoV-2 drives the loss of immune tolerance for IgG leading to rheumatoid arthritis-associated RFs are still emerging. However, more is known about these RFs than RFs induced by other pathogens. Although RFs were first discovered almost a century ago (26) and some unique RF reactivities were reported for rheumatoid arthritis (16–18), the concept of disease-associated RFs with unique IgG reactivities and specific multi-reactivities is new (9). Accordingly, the disease-specific reactivities of RFs induced by the majority of infections are unknown, limiting our mechanistic understanding. However, molecular mimicry (27) and multi-reactivity are likely drivers. Molecular mimicry with viruses like Epstein–Barr virus has been hypothesized to drive autoimmunity in rheumatoid arthritis and other conditions (28). Multi-reactive antibodies are induced by infection, with the beneficial outcome of broadly neutralizing antibodies and the potentially pathogenic outcome of autoreactivity, likely due to defects in B cell tolerance (29–32). Consistent with these phenomena, RFs are known to be multi-reactive with self and pathogen antigens (33, 34).

Antibodies that bind IgG1-131 are particularly interesting when considering mechanisms of immune tolerance loss. The IgM RF that binds IgG1-131 in a citrulline- and homocitrulline-independent manner is multi-reactive with many antigens based on a tripeptide motif, including SARS-CoV-2 spike protein (9). Here we show that IgG1-131 is also bound by IgA post-COVID-19, most prominently in its homocitrulline-containing form (**Figure 3**). IgA from some rheumatoid arthritis participants also binds IgG1-131 in citrulline- and homocitrulline-containing form. Finally, in rheumatoid arthritis, but not COVID-19, IgG binding is strongly elevated exclusively to citrulline- and homocitrulline-containing forms of IgG1-131. These findings suggest that this area of IgG may be particularly susceptible to post-viral autoreactivity due to molecular mimicry and multi-reactivity with potential epitope spreading (35) to homocitrullinated and citrullinated versions of the epitope in rheumatoid arthritis. Molecular mimicry, multi-reactivity, immune dysregulation, or other processes like the recently proposed immune jumping (36) might drive the development of other rheumatoid arthritis-associated RFs.

**Figure 6:**
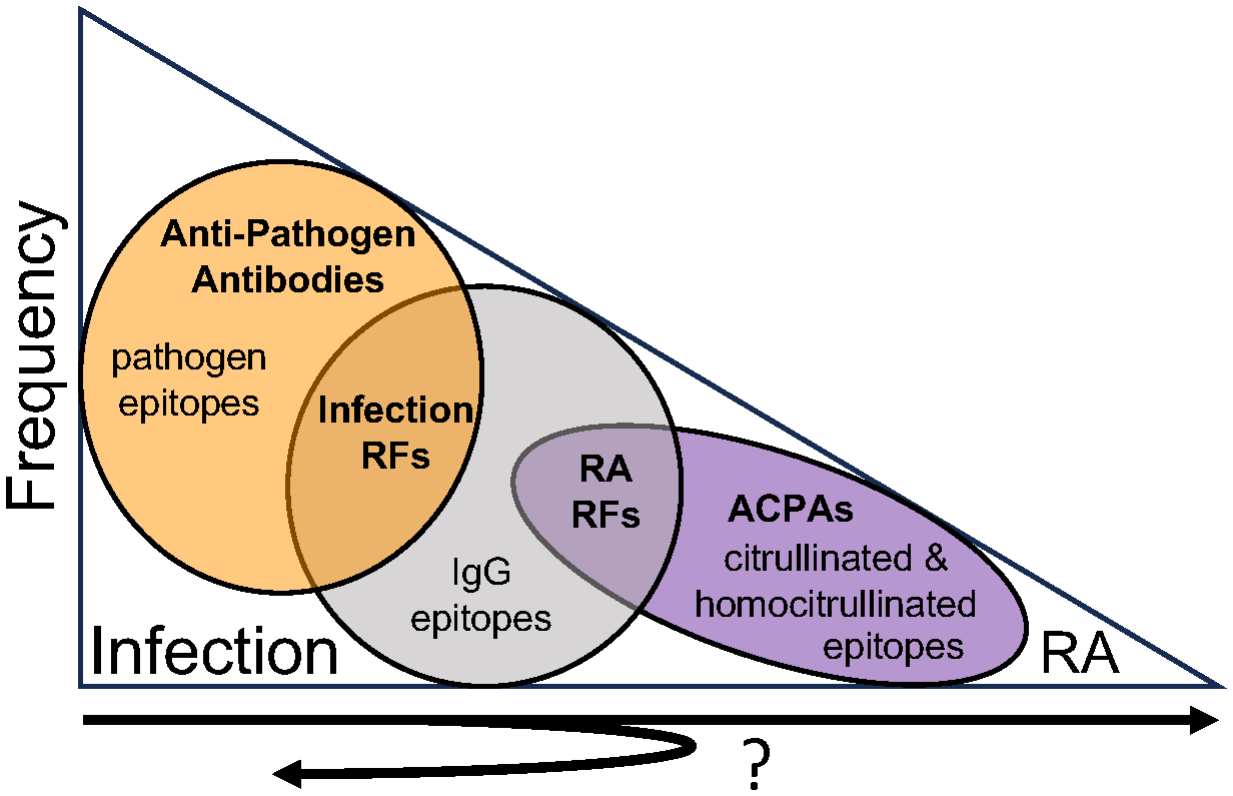
Antibody frequency and reactivity post-infection. Upon infection with pathogens like SARS-CoV-2, the vast majority of individuals generate anti-pathogen antibodies. Fewer individuals generate infection-associated IgM rheumatoid factors (RFs) that bind both pathogens and IgG. Increasingly fewer individuals develop rheumatoid arthritis-associated RFs, multi-reactive anti-citrullinated protein antibodies (ACPAs) as detected by anti-cyclic citrullinated peptide (CCP) testing, and rheumatoid arthritis. It remains unknown if these antibodies represent sequential steps of immune tolerance loss from infection to rheumatoid arthritis, with many individuals demonstrating limited loss of tolerance (i.e. just infection-associated RFs) and progressively fewer individuals experiencing more and more loss of immune tolerance corresponding with additional autoantibodies and ultimately rheumatoid arthritis. Further, it remains unknown if rheumatoid arthritis-associated RFs might resolve as immune tolerance is regained, persist with no clinical consequence, or expand in reactivity over time in some individuals.

Combining our findings with previous work in autoantibodies and rheumatoid arthritis post-COVID-19, the frequency of antibody types that develop can be depicted as a triangle (**Figure 6**). Upon infection with pathogens like SARS-CoV-2, the vast majority of individuals generate anti-pathogen antibodies (37). Fewer individuals, about half in the case of COVID-19, generate infection-associated IgM RFs that bind both pathogen antigens and IgG (9). Fewer individuals generate a repertoire of rheumatoid arthritis-associated RFs (especially of the IgG isotype) and even fewer individuals develop high levels of anti-CCP (38, 39), which often represent multi-reactive ACPAs. Finally, the fewest number of individuals develop rheumatoid arthritis (14, 15). However, it remains unknown if these autoantibodies represent a linked progression of immune tolerance loss, especially given the cross-reactivity, or simply events that occur with different frequencies. Another unknown is how this pathway might vary in response to different pathogens.

There are several limitations to our study. First, our sample size is small, which limits statistical power and generalizability. To partially address this issue, we have noted strong trends in the text. A second limitation is that we evaluate RFs only in response to a single pathogen, again limiting generalizability. A third limitation is that antibody levels were only evaluated five weeks post-COVID-19 and not before COVID-19 or after the five week timepoint. Due to this cross-sectional design, we do not know if rheumatoid arthritis-associated RFs resolve as immune tolerance is restored, persist with or without the development of rheumatoid arthritis, or expand in number and levels on a path towards rheumatoid arthritis. Also, we cannot conclusively determine if rheumatoid arthritis-associated RFs are present post-COVID-19 due to imperfect specificity for rheumatoid arthritis versus early post-viral, rheumatoid arthritis-related loss of immune tolerance, a critical distinction for biomarker development.

Nonetheless, this work is an important step towards understanding post-infection autoimmunity. In combination with our previous work (9), we defined the specificities of RFs post-COVID-19 at a high level of granularity and discovered that some individuals develop moderate levels of some rheumatoid arthritis-associated RFs post-COVID-19. Future research is needed to evaluate rheumatoid arthritis-related RFs and rheumatoid arthritis development in larger populations as well as longitudinally following COVID-19 and other infections to determine if rheumatoid arthritis-associated RFs resolve, persist, or expand in reactivity to include other post-translationally modified epitopes over time. Such studies will shed light on the chronology of immune tolerance loss from viral infection to rheumatoid arthritis as well as reveal if rheumatoid arthritis-associated RFs are specific for rheumatoid arthritis and are potentially useful biomarkers in preclinical rheumatoid arthritis.

## Data Availability

The original contributions presented in the study are included in the article/**Supplementary Material**. Further inquiries can be directed to the corresponding author.
